# Effects of Alkalinity Exposure on Antioxidant Status, Metabolic Function, and Immune Response in the Hepatopancreas of *Macrobrachium nipponense*

**DOI:** 10.3390/antiox13010129

**Published:** 2024-01-21

**Authors:** Shubo Jin, Mingjia Xu, Xuanbin Gao, Sufei Jiang, Yiwei Xiong, Wenyi Zhang, Hui Qiao, Yan Wu, Hongtuo Fu

**Affiliations:** 1 Key Laboratory of Freshwater Fisheries and Germplasm Resources Utilization, Ministry of Agriculture and Rural Affairs, Freshwater Fisheries Research Center, Chinese Academy of Fishery Sciences, Wuxi 214081, China; jinsb@ffrc.cn (S.J.); jiangsf@ffrc.cn (S.J.); xiongyw@ffrc.cn (Y.X.); zhangwy@ffrc.cn (W.Z.); qiaoh@ffrc.cn (H.Q.); wuyan@ffrc.cn (Y.W.); 2 Wuxi Fisheries College, Nanjing Agricultural University, Wuxi 214081, China; xumingjia8562@163.com (M.X.); gaoxuanbin@163.com (X.G.)

**Keywords:** *Macrobrachium nipponense*, hepatopancreas, alkali treatment, metabolic profiling analysis, transcriptome profiling analysis

## Abstract

The oriental river prawn *Macrobrachium nipponense* is an important freshwater economic species in China, producing huge economic benefits. However, *M. nipponense* shows lower alkali tolerance than fish species, thus genetic selection is urgently needed in order to improve alkali tolerance in this species. In the present study, the effects of alkalinity exposure on the hepatopancreas of *M. nipponense* were measured under the alkali concentrations of 0 (control), 4, 8, and 12 mmol/L with the exposure time of 96 h through histological observations, measurement of antioxidant enzymes, metabolic profiling analysis, and transcriptome profiling analysis. The present study identified that the low concentration of alkali treatment (<4 mmol/L) did not result in morphological changes in the hepatopancreas and activity changes in antioxidant enzymes, while high-alkali treatment (>8 mmol/L) damaged the normal structures of the lumen and vacuoles and significantly stimulated the levels of superoxide dismutase, catalase, and total antioxidant capacity, indicating these antioxidant enzymes play essential roles in the protection of the body from the damage caused by the alkali treatment. Metabolic profiling analysis revealed that the main enriched metabolic pathways of differentially expressed metabolites in the present study were consistent with the metabolic pathways caused by environmental stress in plants and other aquatic animals. Transcriptome profiling analysis revealed that the alkali concentration of <8 mmol/L did not lead to significant changes in gene expression. The main enriched metabolic pathways were selected from the comparison between 0 mmol/L vs. 12 mmol/L, and some significantly up-regulated genes were selected from these metabolic pathways, predicting these selected metabolic pathways and genes are involved in the adaptation to alkali treatment in *M. nipponense*. The expressions of Ras-like GTP-binding protein, Doublesex and mab-3 related transcription factor 1a, and Hypothetical protein JAY84 are sensitive to changes in alkali concentrations, suggesting these three genes participated in the process of alkali adaptation in *M. nipponense*. The present study identified the effects of alkalinity exposure on the hepatopancreas of *M. nipponense*, including the changes in antioxidant status and the expressions of metabolites and genes, contributing to further studies of alkali tolerance in this species.

## 1. Introduction

The oriental river prawn, *Macrobrachium nipponense*, is widely distributed in China and other Asian countries [1]. It is an important commercial freshwater prawn species in China with annual production of over two hundred thousand tons, accounting for 5.72% of the total production of freshwater prawns. The main regions for *M. nipponense* culture include Jiangsu Province, Anhui Province, Zhejiang Province, and Jiangxi Province, producing huge economic benefits [2]. The main culture region of *M. nipponense* is in the southeast part of China, while the production in the north part of China is limited. A reasonable reason for this is that the water in the north part of China is mainly saline–alkali water and *M. nipponense* cannot adapt to this water environment.

Alkali tolerance has been identified in many fish and crustacean species (Table 1). The fish species include *Ctenopharyngodon idellus*, *Hypophthalmichthys molitrix*, *Aristichthys nobolis*, *Tribolodon brandti*, and *Gymnocypris przewalskii* [3,4,5]. The crustacean species include *Penaeus chinensis*, *Penaeus vannamei*, and *Palaemon przewalskii* [6,7,8]. Previous study has shown *LC*_50_ values of alkalinity of 27.66 mmol/L at 12 h, 26.94 mmol/L at 24 h, 22.51 mmol/L at 48 h, 15.00 mmol/L at 72 h, and 14.42 mmol/L at 96 h with a safety value of 4.71 mmol/L under conditions of water temperature of (23.1 ± 1.48) °C, pH = (8.9 ± 0.30), salinity of (0.62 ± 0.27), and dissolved oxygen level of (7.2 ± 0.30) mg/L, using Taihu No2 as the research species (a new variety of *M. nipponense* through genetic selection) [9]. Alkalinity tolerance in crustacean species was generally lower than that of fish species. There are extensive saline–alkali water resources in China. However, the alkali tolerance of *M. nipponense* is insufficient to adapt to water environments with high alkali concentrations. Thus, it is important for the sustainable development of the *M. nipponense* industry if the alkali tolerance can be improved in this species. Therefore, studies on the mechanism of alkali tolerance in *M. nipponense* are urgently needed, including the identification of alkali-tolerance-related genes and SNPs.

Transcriptome-profiling analyses have been conducted in many aquatic animals in order to select alkali-tolerance-related genes, including *Leuciscus waleckii* [10], *Lateolabrax maculatus* [11], *Luciobarbus capito* [12], and *Leuciscus waleckii* [13]. These studies suggested that pathways related to stress response and extreme environment adaptation are the main enriched metabolic pathways of differentially expressed genes, including phenylalanine, tyrosine and tryptophan biosynthesis, cell cycle, and DNA replication.

In the present study, we aimed to analyze the effects of alkalinity exposure on the morphological changes in the hepatopancreas and the levels of antioxidants in the hepatopancreas after exposure of the prawns to water environments with different alkali concentrations (0, 4, 8, and 12 mmol/L). Furthermore, the integrated analysis of the transcriptome and metabolome was also performed in order to select genes and metabolites in response to the treatment of alkalinity.

## 2. Materials and Methods

### 2.1. Sample Collection

All of the wild prawns (*M. nipponense*) from the Yangtze River used in the present study were provided by the Dapu *M. nipponense* Breeding Base in Wuxi, China (120°13′44″ E, 31°28′22″ N). A total of 1200 prawns were collected with a body weight of 3.79–4.21 g for males and 2.31–3.14 for females and randomly divided into four groups. The prawns were kept in aerated fresh water with dissolved oxygen content ≥ 6 mg/L for 3 days prior to the alkali treatment. Previous study has identified *LC*_50_ values of alkalinity as 14.42 mmol/L at 96 h in *M. nipponense* [9]. Thus, four alkali concentrations were prepared through adding NaHCO_3_ to the aerated fresh water in the present study, including 0 (control, water without NaHCO_3_), 4, 8, and 12 mmol/L under conditions of water temperature of (28.3 ± 1.26) °C, pH = (7.81–8.32), and dissolved oxygen level of >6.0 mg/L. The alkali concentrations were measured according to the criterion of SC/T9406-2012 [14]. Each alkali concentration was prepared in three tanks, and 100 prawns were maintained in each tank. All prawns were maintained in the different alkali concentrations for 96 h, and then hepatopancreases were collected for histological observations, measurement of antioxidant enzymes, metabolic profiling analysis, transcriptome-profiling analysis, and qPCR analysis. Five hepatopancreases were collected from each alkali concentration and pooled together to form a biological replicate. Eight biological replicates were performed for metabolic profiling analysis, while three biological replicates were performed for the measurement of the activities of antioxidant enzymes, transcriptome-profiling analysis, and qPCR analysis.

### 2.2. Hematoxylin and Eosin (HE) Staining of Hepatopancreas

First, 4% paraformaldehyde was used to fix the tissues used for the histological observations. The hepatopancreases were collected from three individuals of each alkali concentration in order to analyze the morphological changes in the hepatopancreas caused by the alkali treatment. All three hepatopancreases from each concentration were sliced (three biological replicates), and two slices were prepared from each tissue (two technique replicates). The detailed procedures of HE staining have been well described in previous studies [15,16]. Briefly, hepatopancreases were dehydrated in varying ethanol concentrations. The dehydrated hepatopancreases were then transparent and embedded by using different percentages of xylene/wax mixture. The embedded hepatopancreases were finally sliced to 5 µm thickness using a slicer (Leica, Wetzlar, Germany). HE was used to stain the slices for 3–8 min. An Olympus SZX16 microscope (Olympus Corporation, Tokyo, Japan) was used to view the morphological changes.

### 2.3. Measurement of the Activities of Antioxidant Enzymes

The activities of antioxidant enzymes were measured in the hepatopancreas by using commercial kits purchased from the Nanjing Jiancheng Bioengineering Institute, including malondialdehyde (MAD), superoxide dismutase (SOD), catalase (CAT), glutathione (GSH), glutathione peroxidase (GSH-PX), and total antioxidant capacity (T-AOC). All the antioxidant indexes were measured by using a microplate reader (Bio-rad iMark, San Francisco, CA, USA), following the manufacturer’s instructions.

### 2.4. Metabolic Profiling Analysis

Metabolic profiling analysis was performed to select the differentially expressed metabolites (DEMs) in *M. nipponense* caused by the alkali treatment, which were determined by liquid chromatography–mass spectrometry (LC/MS) analysis [17]. The detailed procedures for the metabolic profiling analysis have been well described in a previous published paper [18]. The metabolic profiling was analyzed by an ACQUITY UHPLC system (Waters Corporation, Milford, CT, USA) and an AB SCIEX Triple TOF 5600 System (AB SCIEX, Framingham, MA, USA) in both ESI positive and ESI negative ion modes. The criterion of a seven-fold cross-validation was employed to ensure the robustness and predictive ability of the model, and permutation tests were employed to perform further validation.

### 2.5. Transcriptome-Profiling Analysis

Transcriptome-profiling analysis was performed to select the differentially expressed genes (DEGs) in *M. nipponense* caused by the alkali treatment, which were sequenced by an Illumina Hiseq-2500 sequencing platform. The detailed procedures for the RNA-Seq and analysis have been well described in previous published papers [19,20]. Briefly, the total RNA was extracted from each biological replicate, conducted by using RNAiso Plus Reagent (TaKaRa, San Jose, CA, USA), according to the manufacturer’s instructions. A spectrophotometer (Eppendorf, Hamburg, Germany) was employed to measure the concentration of total RNA. A 2100 Bioanalyzer (Agilent Technologies, Inc., Santa Clara, CA, USA) was employed to measure the integrity of total RNA, and RNA integrity number (RIN) value should be >7.0. A total of 4 µg of total RNA was used to construct the library. The sequencing was conducted by using the Illumina Hiseq-2500 sequencing platform under the parameter of PE150.

Fastp software (version 0.11.5) was employed to remove the low-quality raw reads with the default parameters [21]. The HISAT2 software (version 2.2.1.0) was then employed to map the obtained clean reads to the *M. nipponense* reference genome (GenBank access numbers: GCA_015110555.1 and GCA_015104395.1) [22]. Genes were annotated in the Gene Ontology (GO) (http://www.geneontology.org/, accessed on 15 August 2023) [23], Cluster of Orthologous Groups (COG) (http://www.ncbi.nlm.nih.gov/COG/, accessed on 15 August 2023) [24], and Kyoto Encyclopedia of Genes and Genomes (KEGG) databases (http://www.genome.jp/kegg/, accessed on 15 August 2023) [25], using an E-value of 10^−5^ [19]. Gene expression was calculated using the FPKM method, where FPKM = cDNA fragments/mapped fragments (millions)/transcript length (kb), using HTSeq-count [26]. DESeq2 was used to perform the differential expression analysis [27]. The Benjamini–Hochberg correction method was used to calculate the false discovery rate (FDR) [28] with q-value < 0.05. Fold change >2 was considered to show up-regulated differentially expressed genes (DEGs), and fold change < 0.5 was considered to show down-regulated DEGs.

### 2.6. qPCR Analysis

qPCR was used to measure the expression of DEGs selected from the present study in order to verify the accuracy of RNA-Seq. Previously published studies have described the detailed procedures [29,30]. Briefly, total RNA was extracted from the hepatopancreas of each biological replicate, using the UNlQ-10 Column TRIzol Total RNA Isolation Kit (Sangon, Shanghai, China). A total of 1 μg total RNA from each tissue was used to synthesize the cDNA template, according to the manufacturer’s instructions for the PrimeScript™ RT reagent kit (Takara Bio Inc., Shiga, Japan). The UltraSYBR Mixture (CWBIO, Beijing, China) was used to measure the expression level of each tissue, according to the manufacturer’s instructions. The Bio-Rad iCycler iQ5 Real-Time PCR System (Bio-Rad) was used to conduct the qPCR analysis, which can carry out SYBR Green RT-qPCR assay. Table 2 lists all of the primers used in the present study for qPCR analysis. The eukaryotic translation initiation factor 5A (EIF) has been proven to be a suitable and stable reference gene under various conditions in *M. nipponense* and was used in this study [31]. The 2^−ΔΔCT^ method was used to determine the relative expression levels [32].

### 2.7. Statistical Analysis

SPSS Statistics 23.0 was employed to carry out the statistical analysis in the present study, estimated by one-way ANOVA, followed by Duncan’s multiple range test [29,30]. A probability level of 0.05 was used to indicate significance (*p* < 0.05). The homogeneity of variances was measured in prior to ANOVA (Sig. > 0.05). Meanwhile, a linear regression analysis was performed on each set of data. The linear regression analysis revealed that the residual deviation is close to 1, while the mean residual of each group of data is close to 0, indicating that the residuals of the data are normally distributed and can be analyzed. The confidence intervals were calculated at the 95% level. Quantitative data were expressed as the mean ± SD.

## 3. Results

### 3.1. Survival Rate under Different Alkaline Concentrations

**Figure 1:**
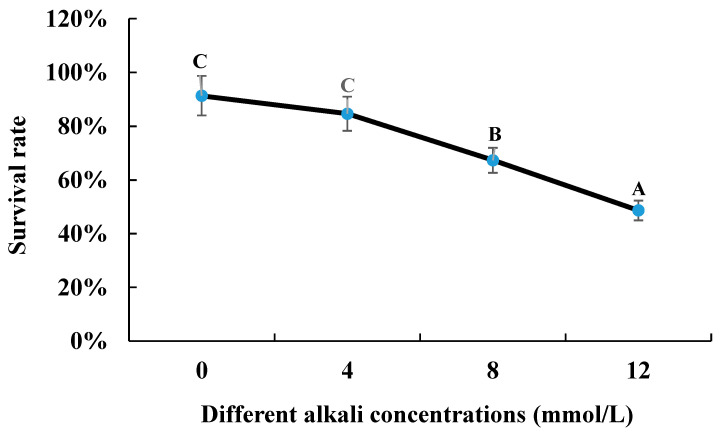
The survival rate of *M. nipponense* under the treatment of different alkali concentrations. Letters indicated the differences of survival rate between different alkali concentrations.

The survival rate gradually decreased with the increase in alkali concentration after the 96 h of alkaline treatment. The survival rate of 0 mmol/L was 91.33%, compared to that of 48.66% under the alkaline concentration of 12 mmol/L (Figure 1). 

### 3.2. Histological Observations

**Figure 2:**
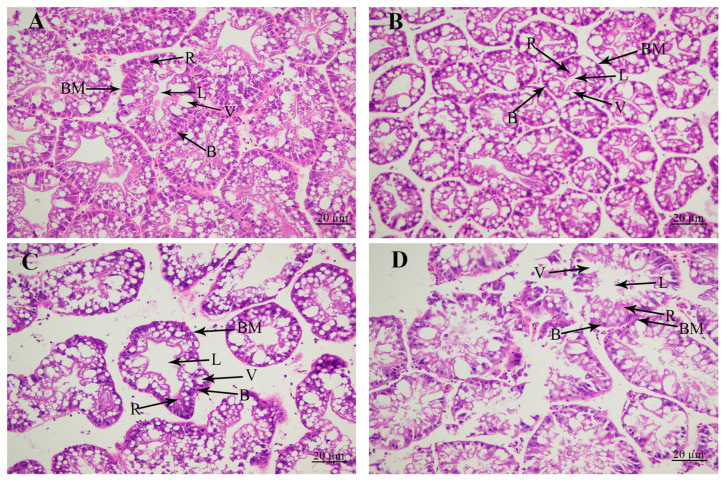
The changes in hepatopancreas under the treatment of different alkali concentrations by histological observations. B: secretory cells of type B; BM: basement membrane; L: lumen; R: storage cells of type R; V: vacuoles. Scale bars = 20 µm. (**A**) The histological observation of hepatopancreas under the alkali concentration of 0 mmol/L; (**B**) the histological observation of hepatopancreas under the alkali concentration of 4 mmol/L; (**C**) the histological observation of hepatopancreas under the alkali concentration of 8 mmol/L; (**D**) the histological observation of hepatopancreas under the alkali concentration of 12 mmol/L.

The morphological changes in the hepatopancreas caused by the alkali treatment were revealed by the histological observations (Figure 2). Histological observations revealed that the hepatopancreas included secretory cells, basement membrane, lumen, storage cells, and vacuoles. The tissue morphology of the hepatopancreas was normal without significant damage at concentrations of 0 mmol/L and 4 mmol/L. However, the alkalinity at the concentration of 8 mmol/L resulted in the increase in the lumen and vacuoles, and secretory cells and storage cells were decreased. When the alkaline concentration reached 12 mmol/L, the lumen and vacuoles of the hepatopancreas were significantly increased, and the basement membrane was severely damaged, affecting the morphology of secretory cells and storage cells in the hepatopancreas.

### 3.3. Measurement of the Activities of Antioxidant Enzymes

**Figure 3:**
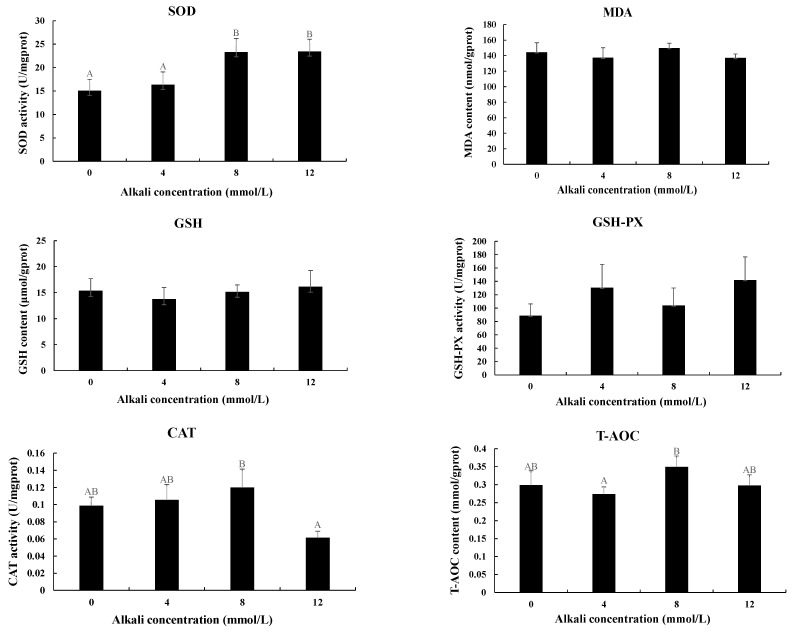
The measurements of the activities of antioxidant enzymes in the hepatopancreas under the treatment of different alkali concentrations. CAT: catalase; GSH: glutathione; GSH-PX: glutathione peroxidase; MDA: malondialdehyde; SOD: superoxide dismutase; T-AOC: total antioxidant capacity. Data are shown as mean ± standard deviation (SD) of tissues from three biological replicates. Capital letters indicated the significant difference of the activities of antioxidant enzymes between different alkali concentrations.

The activities of antioxidant enzymes were also measured in the hepatopancreas after the treatment with different alkali concentrations (Figure 3). The activities of SOD were gradually increased with the increase in alkali concentrations. The activities at the concentrations of 8 mmol/L and 12 mmol/L were significantly higher than those of 0 mmol/L and 4 mmol/L (*p* < 0.05), while the activities between 0 mmol/L and 4 mmol/L and between 8 mmol/L and 12 mmol/L showed no significant difference (*p* > 0.05). The highest activities of CAT and T-AOC were observed at the concentration of 8 mmol/L, which showed a significant difference from those of 12 mmol/L and 4 mmol/L, respectively (*p* < 0.05). However, the activities of MDA, GSH, and GSH-PX showed no difference after the treatment of different concentrations of alkali. Interestingly, all of these six enzymes showed no difference between 0 mmol/L and 4 mmol/L (*p* > 0.05).

### 3.4. Metabolome-Profiling Analysis

**Figure 4:**
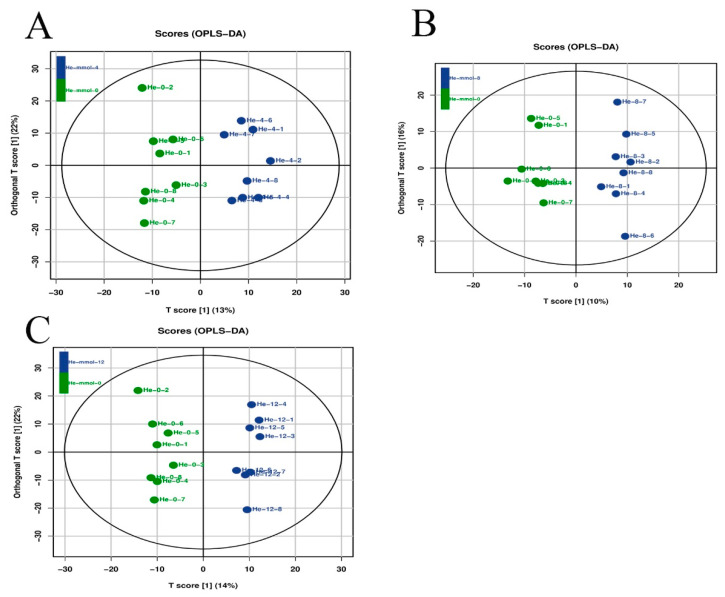
Orthogonal projections of latent structure discriminate analysis (OPLS-DA) of hepatopancreas after the treatment of different alkali concentrations. The LC-MS spectra were used to measure the OPLS-DA score. (**A**) The OPLS-DA analysis of 0 mmol/L vs. 4 mmol/L; (**B**) the OPLS-DA analysis of 0 mmol/L vs. 8 mmol/L; (**C**) the OPLS-DA analysis of 0 mmol/L vs. 12 mmol/L.

Latent structure discriminant analysis was used to analyze the overall quality of the metabolic profiling analysis in the present study (Figure 4), suggesting a robust and reliable model to identify the different metabolic patterns in the hepatopancreas of *M. nipponense* after the treatment of different alkali concentrations.

The differentially expressed metabolites (DEMs) were selected based on the criterion of >2.0 for up-regulated metabolites and <0.5 for down-regulated metabolites. A total of 114 metabolites were differentially expressed between the alkali concentration of 0 mmol/L and 4 mmol/L, of which 85 metabolites were up-regulated and 29 metabolites were down-regulated. Sixty-eight metabolites showed differential expression at the alkali concentrations of 0 mmol/L and 8 mmol/L, including forty-five up-regulated metabolites and twenty-three down-regulated metabolites. A total of 139 DEMs were identified between the alkali concentrations of 0 mmol/L and 12 mmol/L, of which 115 metabolites were up-regulated and 24 metabolites were down-regulated. KEGG analysis revealed that metabolic pathways, biosynthesis of secondary metabolites, biosynthesis of plant secondary metabolites, biosynthesis of amino acids, and microbial metabolism in diverse environments represented the main enriched metabolic pathways of DEMs of all three comparisons in the present study (Table 3).

### 3.5. Transcriptome-Profiling Analysis

A total of 44,084 genes matched the known genes in the *M. nipponense* genome, which is mostly consistent with the number of genes (44,086) in the *M. nipponense* genome. However, 4938 novel isoforms were also predicted in this transcriptome analysis, of which the gene functions need further investigation.

The DEGs were selected based on the criterion of >2.0 for up-regulated genes and <0.5 for down-regulated genes in the present study. A total of 184, 149, and 3949 DEGs were identified in the hepatopancreas between 0 mmol/L vs. 4 mmol/L, 0 mmol/L vs. 8 mmol/L, and 0 mmol/L vs. 12 mmol/L, respectively. Sixty-seven down-regulated DEGs and one hundred and seventeen up-regulated DEGs were identified between 0 mmol/L vs. 4 mmol/L. The comparison between 0 mmol/L vs. 8 mmol/L identified 57 up-regulated DEGs and 92 down-regulated DEGs. A total of 1630 up-regulated DEGs and 2319 down-regulated DEGs were identified between 0 mmol/L vs. 12 mmol/L.

A total of 157, 130, and 3637 DEGs were annotated in the GO database between 0 mmol/L vs. 4 mmol/L, 0 mmol/L vs. 8 mmol/L, and 0 mmol/L vs. 12 mmol/L, respectively. Cells, cell parts, binding, cellular processes, catalytic activity, and metabolic processes were the main enriched functional groups in all of these three comparisons, indicating the genes enriched in these functional groups may play essential roles in the adaptation to alkaline stress in this species (Table 4). 

A total of 32 and 41 DEGs were annotated in the KEGG database between 0 mmol/L vs. 4 mmol/L and 0 mmol/L vs. 8 mmol/L, respectively. Peroxisome was the most enriched metabolic pathway between 0 mmol/L vs. 4 mmol/L, of which five DEGs were enriched. Retinol metabolism, pentose and glucuronate interconversions, and metabolism of xenobiotics by cytochrome P450 with four DEGs were identified as the most enriched metabolic pathways between 0 mmol/L vs. 8 mmol/L. The number of DEGs between 0 mmol/L vs. 12 mmol/L reached 1045, which were annotated in the KEGG database. Endocytosis, RNA transport, protein processing in endoplasmic reticulum, lysosome, ubiquitin mediated proteolysis, ribosome, mTOR signaling pathway, and oxidative phosphorylation represent the most enriched metabolic pathways between 0 mmol/L vs. 12 mmol/L, of which the number of DEGs was ≥40. The main metabolic pathways in each comparison are listed in Table 5.

A total of 25 genes were considered as the strong candidate genes that were predicted to be involved in the mechanism of alkaline tolerance in *M. nipponense*. Three genes were differentially expressed among all of these three comparisons, indicating these three genes are sensitive to changes in alkaline concentrations. These three genes included Ras-like GTP-binding protein (*RaG*), Doublesex and mab-3 related transcription factor 1a (*Dmrt1-a*), and Hypothetical protein JAY84 (*HP-JAY84*). The other 22 genes were significantly differentially expressed between 0 mmol/L vs. 12 mmol/L, which were enriched in the main enriched metabolic pathways (Table 6).

### 3.6. qPCR Analysis

**Figure 5:**
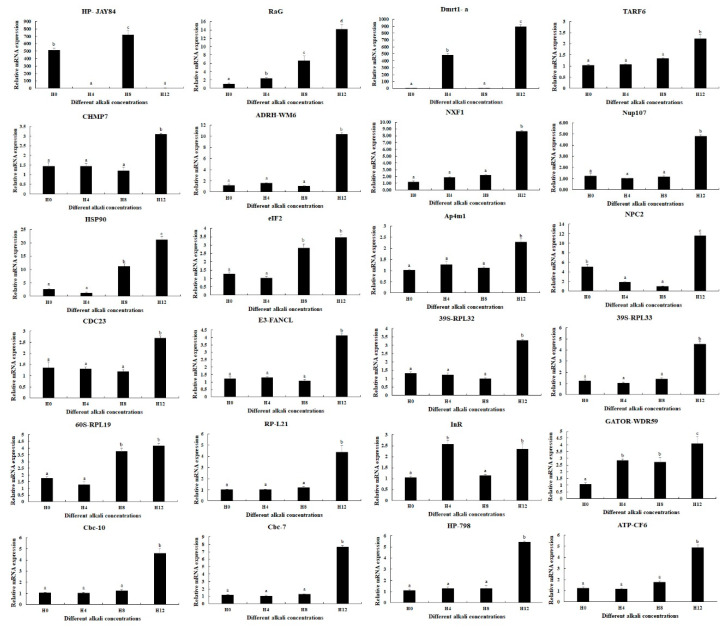
qPCR analyses of the expressions of DEGs in the hepatopancreas under the treatment of different alkali concentrations. Data are shown as mean ± standard deviation (SD) of tissues from three biological replicates. Letters indicate a significant difference in the expressions of DEGs between different alkali concentrations.

qPCR analyses were used to verify the expressions of DEGs selected from this study (Figure 5). qPCR analyses showed the same expression trends as RNA-Seq. *RaG*, *Dmrt1-a*, and *HP-JAY84* showed differential expressions in all three comparisons (0 mmol/L vs. 4 mmol/L, 4 mmol/L vs. 8 mmol/L, and 8 mmol/L vs. 12 mmol/L) (*p* < 0.05), of which *RaG* was gradually increased with the increase in alkali concentration. Interestingly, qPCR analysis also identified that the *InR* expressions were differentially expressed between all three comparisons (*p* < 0.05). The expressions of fifteen DEGs reached the peak at the alkali concentration of 12 mmol/L (*p* < 0.05), while the expressions at 0 mmol/L, 4 mmol/L and 8 mmol/L remained stable (*p* > 0.05). Two DEGs (*eIF2* and *60S-RPL19*) showed higher expressions at 8 mmol/L and 12 mmol/L than at 0 mmol/L and 4 mmol/L (*p* < 0.05), while the expressions showed no difference between 0 mmol/L and 4 mmol/L and between 8 mmol/L and 12 mmol/L (*p* > 0.05). The expressions of *Hsp90* and *GATOR-WDR59* gradually increased from 0 mmol/L to 12 mmol/L, while the expression showed no significant difference between 0 mmol/L and 4 mmol/L for *Hsp90* and between 4 mmol/L and 8 mmol/L for *GATOR-WDR59* (*p* > 0.05). 

## 4. Discussion

Previous study has identified that the alkaline *LC*_50_ at 12 h, 24 h, 48 h, 72 h, and 96 h in juvenile prawns of “Taihu No2” (a new variety of *M. nipponense*, selected through the hybridization of *M. nipponense* and *M. hainanense*) were 27.66 mmol/L, 26.94 mmol/L, 22.51 mmol/L, 15.00 mmol/L, and 14.42 mmol/L, respectively [9]. Compared with other prawn or shrimp species, juvenile *M. nipponense* showed stronger alkali resistance and can be cultured in appropriate saline and alkali water. However, the tolerance of carbonate alkalinity of this species is dramatically lower than those of freshwater fish species. Thus, the long-term goal is to find out the mechanism of alkali tolerance in *M. nipponense* in order to culture a new strain of this species with stronger alkali tolerance. In the present study, we investigated the effects of different alkali concentrations on the hepatopancreas of *M. nipponense* through histological observations, measuring the activities of antioxidant enzymes, and performing metabolic profiling analysis and transcriptome-profiling analyses in the hepatopancreas.

The survival rate of *M. nipponense* gradually decreased from 0 mmol/L (91.33%) to 48.33% under the concentration of 12 mmol/L after 96 h of alkali treatment. Previous study has shown that the *LC*_50_ value of alkali treatment at 96 h was 14.42 mmol/L, using juvenile “Taihu No2” as the research species [9]. In the present study, over half of the prawns were dead under the alkali concentration of 12 mmol/L after 96 h of treatment. The above results indicated that “Taihu No2” showed stronger abilities to resist the stress of alkali treatment than Yangtze River wild populations, or stronger abilities to resist the stress of alkali treatment were observed in the juvenile prawns compared to adult prawns. 

Some previous publications have identified the effects of alkali treatment on the morphological changes in gills in aquatic animals [33,34,35,36], while related reports on the morphological changes in the hepatopancreas are rare. Alkali treatment leads to the detachment of the basement membrane of liver tubules from epithelial cells in *Eriocheir sinensis* [37]. In the present study, alkali treatment resulted in the significant damage to the lumen, vacuoles, secretory cells, and storage cells, thus affecting the normal physiological functions of the hepatopancreas. 

The measurement of antioxidant enzymes has been widely used to analyze the effects of stress on the behaviors of prawns [38,39]. The effects of alkali stress on antioxidant enzymes have been widely analyzed in many plants [40,41,42], while the study of the effects on aquatic animals is rare. A pH of 7.8 stimulated the transcript levels of CAT and GPx and the activity of GPx, while strong alkalization (pH 8.8) has negative effects on the activities of antioxidant enzymes, suggesting alkaline exposure has more harmful effects on antioxidant activity in the liver of hybrid tilapia than acidic exposure [43]. The activities of SOD reached the peak at 3 days in the liver of *Gymnocypris przewalskii* after alkaline treatment at concentrations of 32 mmol/L and 64 mmol/L [5]. The activities of SOD and CAT gradually increased and then decreased to a normal level in the liver of *Triplophysa dalaica* after the alkaline treatment [44]. In *E. sinensis*, the activity of T-AOC was significantly increased after the alkali treatment, while SOD showed no difference between the alkali-treated group and control group [37]. Alkali treatment stimulates the production of excessive free oxygen radicals in animals, and thus antioxidant enzymes are responsible for the elimination of the effects of these free oxygen radicals [45]. In the present study, the activities of all of the tested antioxidants showed no difference between 0 mmol/L and 4 mmol/L, indicating the alkaline concentration of 4 mmol/L did not result in changes in the antioxidative stress. In addition, alkali stress did not result in an increase in MDA, GSH, or GSH-PX levels, while the levels of SOD, CAT, and T-AOC were increased, indicating SOD, CAT, and T-AOC play essential roles in the response of *M. nipponense* to acute alkali stress. However, the role of the antioxidative defense system in the adaptive mechanism to alkali stress needs to be further investigated in *M. nipponense* through chronic exposure experiments. 

Metabolic pathways, biosynthesis of secondary metabolites, biosynthesis of amino acids, and microbial metabolism in diverse environments have been identified as the main enriched metabolic pathways of DEMs when environmental stress occurs in plants and aquatic animals [46,47,48,49], which is consistent with the results of the present study. Secondary metabolites are natural products which show a restricted taxonomic distribution. Biosynthesis of secondary metabolites has been a hot research topic recently because they have positive effects on health [50,51]. Amino acids are essential substrates for the synthesis of many biologically active substances, playing essential roles in the maintenance of normal physiological and nutritional status in animals [52]. The present study predicted that biosynthesis of secondary metabolites and biosynthesis of amino acids significantly regulated the response to alkali stress in *M. nipponense*.

In the present study, only 184 and 149 genes were differentially expressed between 0 mmol/L and 4 mmol/L and between 0 mmol/L and 8 mmol/L, respectively. This indicated that a low concentration of alkali treatment did not result in significant changes in gene expression. A total of 3949 genes were identified to be differentially expressed between 0 mmol/L and 12 mmol/L, and endocytosis, RNA transport, protein processing in endoplasmic reticulum, lysosome, ubiquitin mediated proteolysis, ribosome, mTOR signaling pathway, and oxidative phosphorylation were the most enriched metabolic pathways of DEGs. 

Endocytosis is a cellular process which has been reported to be involved in the regulation of cell signaling and the mediation of receptor internalization and nutrient uptake. The endocytic vesicle usually fuses with the early endosome after endocytosis, which accepts newly endocytosed material, serving as a sorting station that directs incoming proteins and lipids to their final destination [53]. TNF receptor-associated factor 6 (TRAF6) is a kind of ubiquitin-ligase, playing an important role in inflammation and immune response. TRAF6 has been identified as a transduction factor, involved in the activation of receptor activator of nuclear factor κB ligand (RANKL), RANK, NFATcl, and lipopolysaccharide signaling [54,55]. Lysosomes mediate a broad range of fundamental processes, including plasma membrane repair, signaling, secretion, and energy metabolism, which has significant implications for health and disease [56,57]. NPC intracellular cholesterol transporter (NPC) is an essential gene in lysosomes, which has been identified to be involved in mitochondrial dysfunction and mTOR suppression [58,59]. Ubiquitin-mediated protein degradation is one of the important mechanisms of protein degradation in cells, playing essential roles in the regulation of various cellular biological processes, including cell cycle, signal transduction, DNA repair, and immune response [60,61]. Ubiquitin E3 ligases (E3) have functions in the reorganization of the target protein, playing essential roles in the mediation of the covalent linkage between target and ubiquitin moieties. These ligases promote target specificity and uniqueness in the process of ubiquitination [62,63]. In the present study, endocytosis, lysosome, and ubiquitin-mediated proteolysis are significantly changed after the alkalinity exposure, mainly functioning in the recognition and digestion of damaged or aged cells caused by the exposure to alkalinity. The alkali concentration of 12 mmol/L significantly stimulated the expressions of TRAF6, NPC2, and E3 FANCL, indicating these genes are involved in the regulation of alkali tolerance in this species.

The endoplasmic reticulum (ER) is an organelle, and proteins are folded with the help of lumenal chaperones in the ER. Newly synthesized peptides are glycosylated in the ER. Correctly folded proteins are packaged into transport vesicles and transferred to the Golgi complex. Misfolded proteins are retained within the ER lumen and finally degraded [64,65]. Heat shock protein 90 (*HSP90*) proteins regulate the process of protein folding, signal transduction, protein degradation, and morphologic evolution. *HSP90* plays essential roles in folding newly synthesized proteins or stabilizing and refolding denatured proteins after stress [66,67]. Eukaryotic translation initiation factor 2 (*eIF2*) is a key protein involved in translation initiation of eukaryotic cells. It plays essential roles in the conversion of eIF2-GDP (inactive state of eIF2) into eIF2-GTP (active state of eIF2) during the process of translation initiation [68,69]. Ribosomes regulate the process of RNA translation into protein and can obtain the genetic information from messenger RNA and convert it into amino acid sequences to synthesize proteins [70,71]. Ribosomal proteins (RPs) are used to synthesize the ribosome. RPs are highly conserved proteins involved in translational control and cellular homeostasis [72]. Thus, protein processing in endoplasmic reticulum and ribosomes were suggested to participate in the regulation of alkali tolerance through ensuring the accuracy of protein synthesis in *M. nipponense* after the exposure to alkalinity. The significantly up-regulated genes from these two metabolic pathways, including *39S-RPL32*, *39S-RPL33*, *60S-RPL19*, *HSP90*, and *eIF2*, possibly promoted protein processing, which contributed to the adaptation to alkali stress in *M. nipponense*.

Oxidative phosphorylation is the main reaction to produce ATP in wild organisms [73]. Cellular respiration is an important process to produce energy in most eukaryotic organisms [74,75,76]. The cytochrome bc1 complex (Cbc) is an essential component of cellular respiration, promoting the generation of ATP [77]. Adenosine triphosphate (ATP) synthase promotes the production of ATP in cells. ATP synthase-coupling factor 6 (*ATP-CF6*) is released from the vascular endothelial cells and was considered as a cardiovascular therapeutic target through inhibiting prostacyclin synthesis and promoting nitric oxide (NO) synthesis [78]. In addition, ATP synthase-coupling factor 6 was identified to inhibit the JAK1-STAT6 signaling pathway and thus suppress male-predominant HCC [79]. Thus, the changes in oxidative phosphorylation in the present study were predicted to regulate the process of alkali tolerance through providing ATP in *M. nipponense*. Furthermore, *Cbc-7*, *Cbc-10*, and *ATP-CF6* were significantly up-regulated under alkali exposure in *M. nipponense*, which showed a positive response to the alkali stress.

Three genes were differentially expressed among all three comparisons, predicting these three genes play essential roles in the mechanism of alkali tolerance of *M. nipponense*, including hypothetical protein JAY84_18770, Ras-like GTP-binding protein, and *DMRT1-a*. Previous study identified that bacterial GTP-binding proteins are a key factor in the regulation of protein biosynthesis and protein secretion [80]. The member of the ras superfamily of GTP-binding proteins act as molecular binary switches, which were identified to be involved in the various cellular processes of an organism, especially for cell growth [81,82]. *DMRT1-a* is a transcription factor which was identified to regulate the process of male sex determination and differentiation. The main functions for *DMRT1-a* included the controlling of testis development and germ cell proliferation, which can act both as a transcription repressor and activator [83,84]. 

The qPCR verification of DEGs was generally consistent with those of RNA-Seq, indicating the accuracy of RNA-Seq. qPCR analyses revealed that the expression of four DEGs was sensitive to the changes in alkali concentrations, especially that of *RaG*, of which the expression was increased with the increase in alkali concentration, indicating these four genes play essential roles in the protection of the body from the damage caused by alkali treatment. In addition, the other tested DEGs showed the highest expressions at the alkali concentration of 12 mmol/L, and slightly changed between 0 mmol/L, 4 mmol/L, and 8 mmol/L, indicating only a high alkali concentration can stimulate significant changes in gene expressions and these genes are involved in the process of alkali tolerance in *M. nipponense*.

## 5. Conclusions

In conclusion, the results of the present study indicated that the death rate of adult wild *M. nipponense* was increased with the increase in alkali concentration. Low-concentration of alkali treatment (<4 mmol/L) did not result in changes in histology and antioxidant enzymes, while higher alkali concentrations stimulated the activities of SOD, CAT, and T-AOC, indicating these enzymes play essential roles in the protection of the body from the damage of alkali treatment. Furthermore, only the alkali concentration of 12 mmol/L led to significant changes in gene expressions, and endocytosis, RNA transport, protein processing in endoplasmic reticulum, lysosome, ubiquitin-mediated proteolysis, ribosome, mTOR signaling pathway, and oxidative phosphorylation represented the most enriched metabolic pathways. Endocytosis, lysosome, and ubiquitin-mediated proteolysis are immune-related metabolic pathways, which protect the body from the damage of alkali treatment and degrade damaged or aged cells. Protein processing in the endoplasmic reticulum and ribosome promoted protein synthesis. Oxidative phosphorylation produces ATP to support the adaptation to alkali treatment in this species. Interestingly, qPCR analyses revealed that four genes were differentially expressed among all three comparisons, predicting these genes were sensitive to the changes in alkali concentration, including *HP-JAY84*, *RaG*, *Dmrt1-a*, and *InR*. The present study identified the changes in antioxidant status, morphology, metabolites, and genes in the hepatopancreas of *M. nipponense* caused by the alkalinity exposure, providing valuable evidence to find out the mechanism of alkali adaptation in this species.

## Figures and Tables

**Table 1:** Alkali tolerance in fishes and crustaceans.

Species	*LC*_50_ Value at 24 h (mmol/L)	Safe Alkali Value (mmol/L)
*Ctenopharyngodon idellus*	82.2	
*Hypophthalmichthys molitrix*	95	
*Aristichthys nobolis*	65.7	
*Tribolodon brandti*	89.31	18.79
*Gymnocypris przewalskii*		64
*Penaeus chinensis*	3.28	
*Penaeus vannamei*	12.40	
*Palaemon przewalskii*		3.5
*Macrobrachium nipponense*	14.42	4.71

**Table 2:** Primers used for the qPCR validation in the present study.

Gene	Forward Primer	Reverse Primer	Efficiency (%)	Product Size (bp)
*HP-JAY84*	CGCTCTAGATCCGTGAGCAG	ACGAGGCCAGAAACTCTTGG	95.4	232
*RaG*	GTCCTCAAGATCGTGGCTGT	TGCATCTGTCGAAACCCTCC	97.1	196
*Dmrt1- a*	GTTGGCTTCGTCCCAGAAGA	TGATCACACTCCACGCTGAC	94.8	185
*TARF6*	TGCTCCATAGTCCGGCATTT	GCAGATCTGGCTTGCTTACC	101.5	240
*CHMP7*	ATTCCGCAGTGGTGTAGAGG	GAGCTCACTCCCGAAGCAAG	99.2	144
*ADRH-WM6*	GGTCCAGGAGAGATTCGACG	CCACACAAAATGCAGCCACA	97.9	117
*NXF1*	TAGGACACCACTTGCTGCTG	TGCATTCGCTTGTCTGAGGT	102.5	153
*Nup107*	AGGAGGAAACTGGCTCTCCA	TCCAGCGATCAAGTTCACCC	97.5	150
*Hsp90*	ATGCCCGAGGAACCAATGAC	AGAGCTCCTTGCCAGATTCG	98.2	208
*eIF2*	TGGAAAGACCGAACCAGTCG	AAAAGCTCCCCTACGTGTCG	103.6	162
*Ap4m1*	TGGAATGGGCACAGTATCACC	CCTCCAGAGTCTACAAGCCG	96.8	201
*NPC2*	CTCTGCTACAGCCTTCCAGG	TTCGACTCCCTTGCAAGCAT	97.1	232
*CDC23*	AGGCTCAGAAATTGCGACCA	GGCACGTTCACCTTCACCTA	97.4	189
*E3-FANCL*	GTGGCAATGAAGAATGGGGC	GTCTGCTATTCGGAAGCCCA	98.6	150
*39S-RPL32*	TGAGCATGAAGTCTTTCCCGT	CAGTCTGCGAGAAAACCACTG	101.5	121
*39S-RPL33*	GGCCAACGTTTTTCGATCTGG	AAAGACGCACACCTGACGGA	97.8	199
*60S-RPL19*	CAATGCTCGTGCCAAGATGT	TCTGCCTTCCTTTGGGCAAC	95.3	105
*RP-L21*	GAGACGCCACAACTCAAGGA	TTCCGTCTACCCCTTCCACT	104.8	122
*InR*	TCCTCGGTGCCTCAAGAAAC	CCACTGCAGACCTCGAATGT	101.9	173
*GATOR-WDR59*	CACATCCATCCACCCCTGTG	ACAGCCTGTTGGGCATTCAT	97.6	263
*Cbc-10*	CCTCTGGAGTGCAATGGGAG	TTGCTGCTGAACCCAGTCTC	99.7	131
*Cbc-7*	AGGAGGAAAGTCGAAAGCCG	AGATGACGAGAAGCACTGGC	99.6	141
*HP-798*	GACGTTCTTCGCACACTTCA	TCATGCGTTCCGTTTCCAGA	102.4	113
*ATP-CF6*	CAAGGTTGCTCGCCAGTATG	TTTTGCAAACAGTTCAGGTGGT	95.6	120
*EIF*	CATGGATGTACCTGTGGTGAAAC	CTGTCAGCAGAAGGTCCTCATTA	98.5	157

**Table 3:** The main metabolic pathways of DEMs.

Metabolic Pathways (0 vs. 4)	DEMs	Metabolic Pathways (0 vs. 8)	DEMs	Metabolic Pathways (0 vs. 12)	DEMs
Metabolic pathways	44	Metabolic pathways	38	Metabolic pathways	59
Biosynthesis of secondary metabolites	21	Biosynthesis of secondary metabolites	13	Biosynthesis of secondary metabolites	31
Biosynthesis of plant secondary metabolites	14	Microbial metabolism in diverse environments	12	Biosynthesis of amino acids	18
Biosynthesis of amino acids	12	Biosynthesis of plant secondary metabolites	11	Microbial metabolism in diverse environments	16
Microbial metabolism in diverse environments	11	Biosynthesis of cofactors	7	Biosynthesis of plant secondary metabolites	18
Protein digestion and absorption	11	Nucleotide metabolism	7	Central carbon metabolism in cancer	13
Aminoacyl-tRNA biosynthesis	9	Biosynthesis of amino acids	6	Biosynthesis of cofactors	12
Central carbon metabolism in cancer	9	Carbon metabolism	6	Protein digestion and absorption	12
Mineral absorption	8	Cysteine and methionine metabolism	6	ABC transporters	10
ABC transporters	8	Biosynthesis of plant hormones	6	Carbon metabolism	10
2-Oxocarboxylic acid metabolism	7	Biosynthesis of alkaloids derived from histidine and purine	6	Aminoacyl-tRNA biosynthesis	10
Glucosinolate biosynthesis	7	Purine metabolism	6	2-Oxocarboxylic acid metabolism	10
Biosynthesis of various plant secondary metabolites	7	D-Amino acid metabolism	5	Glycine, serine, and threonine metabolism	9
D-Amino acid metabolism	6	Taste transduction	5	D-Amino acid metabolism	9
Biosynthesis of plant hormones	6	Glyoxylate and dicarboxylate metabolism	5	Mineral absorption	8

**Table 4:** The main functional groups of DEGs by GO analysis.

0 mmol/L vs. 4 mmol/L	0 mmol/L vs. 8 mmol/L	0 mmol/L vs. 12 mmol/L
Binding	Cell	Cell
Catalytic activity	Cell part	Cell part
Cellular process	Binding	Cellular process
Cell	Cellular process	Binding
Cell part	Catalytic activity	Metabolic process
Metabolic process	Metabolic process	Organelle
Organelle	Membrane	Catalytic activity
Membrane	Organelle	Biological regulation
Membrane part	Membrane part	Organelle part
Extracellular region	Biological regulation	Developmental process
Multicellular organismal process	Response to stimulus	Multicellular organismal process
Developmental process	Organelle part	Membrane
Response to stimulus	Developmental process	Cellular component organization or biogenesis
Biological regulation	Multicellular organismal process	Response to stimulus
Localization	Localization	Protein-containing complex

**Table 5:** The main metabolic pathways of DEGs by KEGG analysis.

Metabolic Pathways (0 vs. 4)	DEGs	Metabolic Pathways (0 vs. 8)	DEGs	Metabolic Pathways (0 vs. 12)	DEGs
Peroxisome	5	Retinol metabolism	4	**Endocytosis**	59
Arginine and proline metabolism	4	Pentose and glucuronate interconversions	4	**RNA transport**	53
Fatty acid degradation	3	**Metabolism of xenobiotics by cytochrome P450**	4	**Protein processing in endoplasmic reticulum**	48
Drug metabolism—other enzymes	3	Glycerophospholipid metabolism	3	**Lysosome**	44
Ascorbate and aldarate metabolism	3	Drug metabolism—cytochrome P450	3	**Ubiquitin mediated proteolysis**	43
**Metabolism of xenobiotics by cytochrome P450**	2	Glutathione metabolism	3	**Ribosome**	43
Tryptophan metabolism	2	Fructose and mannose metabolism	3	**mTOR signaling pathway**	42
Pentose and glucuronate interconversions	2	**Phagosome**	3	**Oxidative phosphorylation**	40
Drug metabolism—cytochrome P450	2	**Pyruvate metabolism**	3	**Ribosome biogenesis in eukaryotes**	39
**Pyruvate metabolism**	2	**Citrate cycle (TCA cycle)**	2	**Amino sugar and nucleotide sugar metabolism**	37

Note: The metabolic pathways related to oxidative stress and cellular organization are bolded.

**Table 6:** The main DEGs from the transcriptome-profiling analysis.

Gene	Accession Number	Species	Fold Change		
			0 vs. 4	4 vs. 8	8 vs. 12
Hypothetical protein (*HP-JAY84*)	JAY84_18770	*Candidatus Thiodiazotropha*	0.004	292.04	0.009
Ras-like GTP-binding protein (*RaG*)	XP_053656102.1	*Cherax quadricarinatus*	2.35	6.96	12.91
Doublesex and mab-3 related transcription factor 1a (*Dmrt1-a*)	QDE10512.1	*Macrobrachium rosenbergii*	91.77	0.10	14.32
Gene	Accession number	Species	Metabolic pathway		Fold change
					0 vs. 12
TNF receptor associated factor 6 (*TARF6*)	ASM46956.1	*Macrobrachium nipponense*	Endocytosis		3.58
Charged multivesicular body protein 7 (*CHMP7*)	XP_027209977.1	*Penaeus vannamei*	Endocytosis		6.92
ATP-dependent RNA helicase WM6 (*ADRH-WM6*)	RXG50776.1	*Armadillidium vulgare*	RNA transport		6.15
Ribonuclease P protein subunit p29	XP_027220920.1	*Penaeus vannamei*	RNA transport		12.21
Nuclear RNA export factor 1 (*NXF1*)	XP_045623594.1	*Procambarus clarkii*	RNA transport		4.32
Nuclear pore protein Nup107 (*Nup107*)	XP_027211930.1	*Penaeus vannamei*	RNA transport		4.76
Hsp90 protein	ROT76137.1	*Penaeus vannamei*	Protein processing in endoplasmic reticulum		3.97
Eukaryotic translation initiation factor 2 (*eIF2*)	XP_053634587.1	*Cherax quadricarinatus*	Protein processing in endoplasmic reticulum		3.68
AP-4 complex subunit mu-1-like isoform X2 (*Ap4m1*)	XP_027222938.1	*Penaeus vannamei*	Lysosome		7.11
NPC intracellular cholesterol transporter 2 (*NPC2*)	XP_047502679.1	*Penaeus chinensis*	Lysosome		6.96
Cell division cycle protein 23 (*CDC23*)	XP_027236079.1	*Penaeus vannamei*	Ubiquitin-mediated proteolysis		4.53
E3 ubiquitin-protein ligase FANCL (*E3-FANCL*)	XP_027214794.1	*Penaeus vannamei*	Ubiquitin-mediated proteolysis		48.84
39S ribosomal protein L32 (*39S-RPL32*)	XP_027217747.1	*Penaeus vannamei*	Ribosome		14.32
39S ribosomal protein L33 (*39S-RPL33*)	XP_047997684.1	*Leguminivora glycinivorella*	Ribosome		8.11
60S ribosomal protein L19 (*60S-RPL19*)	XP_027212740.1	*Penaeus vannamei*	Ribosome		10.41
Ribosomal prokaryotic L21 protein (*RP-L21*)	XP_042878336.1	*Penaeus japonicus*	Ribosome		8.51
Insulin-like receptor (*InR*)	XP_027218065.1	*Penaeus vannamei*	mTOR signaling pathway		15.35
GATOR complex protein WDR59 (*GATOR-WDR59*)	XP_027223649.1	*Penaeus vannamei*	mTOR signaling pathway		4.56
Cytochrome b-c1 complex subunit 10 (*Cbc-10*)	KZC10939.1	*Dufourea novaeangliae*	Oxidative phosphorylation		4.53
Cytochrome b-c1 complex subunit 7 (*Cbc-7*)	XP_027231282.1	*Penaeus vannamei*	Oxidative phosphorylation		7.84
Hypothetical protein L798_02749 (*HP-798*)	KDR07695.1	*Zootermopsis nevadensis*	Oxidative phosphorylation		5.21
ATP synthase-coupling factor 6 (*ATP-CF6*)	XP_042857694.1	*Penaeus japonicus*	Oxidative phosphorylation		4.66

## Data Availability

The raw data of the present study have been submitted to NCBI with the accession numbers SRX22243687–SRX22243698 and MetaboLights with the accession number MTBLS8831. All other data are contained within the main manuscript.
